# Consultations with Irregular Practitioners

**Published:** 1850-11

**Authors:** 


*Consultations with Irregular Practitioners.*—The Erie (Penn.) Gazette, contains an able address to the public, by a Committee appointed by the County Medical Society, giving the reasons for a resolution adopted by the Society prohibiting consultations with irregular practitioners. The Committee consisted of Drs. Flint, Beebe, Vosburg, Miller, and Perkins. The merits of the subject are, of course, well understood by the Profession, but very imperfectly by a portion of the public ; and hence the propriety of explaining the line of conduct which it appears to have been necessary for the Erie County Society to prescribe for its members.

